# Prostate Cancer Stem Cells and Nanotechnology: A Focus on Wnt Signaling

**DOI:** 10.3389/fphar.2017.00153

**Published:** 2017-03-28

**Authors:** Wei Qin, Yongjiang Zheng, Bin-Zhi Qian, Meng Zhao

**Affiliations:** ^1^The Third Affiliated Hospital, Zhongshan School of Medicine, Sun Yat-sen UniversityGuangzhou, China; ^2^Key Laboratory for Stem Cells and Tissue Engineering, Ministry of Education, Sun Yat-sen UniversityGuangzhou, China; ^3^Edinburgh Cancer Research UK Centre and MRC University of Edinburgh Centre for Reproductive Health, University of EdinburghEdinburgh, UK; ^4^Department of Pathophysiology, Zhongshan School of Medicine, Sun Yat-sen UniversityGuangzhou, China

**Keywords:** prostate cancer, prostate cancer stem cell, Wnt signaling, nanotechnology, cancer therapy

## Abstract

Prostate cancer is the most common cancer among men worldwide. However, current treatments for prostate cancer patients in advanced stage often fail because of relapse. Prostate cancer stem cells (PCSCs) are resistant to most standard therapies, and are considered to be a major mechanism of cancer metastasis and recurrence. In this review, we summarized current understanding of PCSCs and their self-renewal signaling pathways with a specific focus on Wnt signaling. Although multiple Wnt inhibitors have been developed to target PCSCs, their application is still limited by inefficient delivery and toxicity *in vivo*. Recently, nanotechnology has opened a new avenue for cancer drug delivery, which significantly increases specificity and reduces toxicity. These nanotechnology-based drug delivery methods showed great potential in targeting PCSCs. Here, we summarized current advancement of nanotechnology-based therapeutic strategies for targeting PCSCs and highlighted the challenges and perspectives in designing future therapies to eliminate PCSCs.

## Introduction

Prostate cancer continues to be the most frequently diagnosed cancer in males and the third leading malignancy of cancer-related deaths in the USA (Siegel et al., 2017). Advanced/metastatic prostate cancer has been the major clinical challenge for prostate cancer. Recently, several new treatments have led to significant improvement of overall survival. These include novel androgen receptor pathway inhibitors abiraterone acetate (de Bono et al., 2011; Ryan et al., 2013) and enzalutamide (Scher et al., 2012; Beer et al., 2014), chemotherapy drugs taxanes, docetaxel and cabazitaxel (de Bono et al., 2010), an immunotherapeutic agent Sipuleucel-T (Kantoff et al., 2010), and a bone targeting alpha-emitting radionuclide, radium-223 chloride (Parker et al., 2013; Gillessen et al., 2015). However, resistance and recurrence still persists, which limits patient benefit.

Although still controversial, the resistant to the current treatment (hormonal therapy, chemotherapy, or radiotherapy) can be at least partially explained by the existence of prostate cancer stem cells (PCSCs). These cells can self-renew to initiate tumor *in vivo* in severe combined immunodeficient (SCID) mice (Hurt et al., 2008). PCSCs usually have low or undetectable androgen receptor expression that can lead to the failure of androgen deprivation therapy (hormonal therapy), the standard primary treatment for advanced prostate cancer (Lee et al., 2013; Di Zazzo et al., 2016). The slow growth rate of PCSCs allows them to survive routine chemotherapy and radiotherapy which are designed to attack actively dividing neoplastic cells. Moreover, PCSCs are highly resistant to drugs and toxins through a variety of mechanisms including enhanced drug efflux, expression of anti-apoptosis genes, and active DNA repair (Ni et al., 2014). The surviving PCSCs can regenerate the bulk of the tumor, or give rise to distant metastasis (Hurt et al., 2008; Salvatori et al., 2012; Shiozawa et al., 2016). Therefore, therapeutic strategies that specifically target PCSCs may eradicate tumors more effectively and reduce the risk of relapse and metastasis. PCSCs use various signaling pathways to maintain their self-renewal and differentiation, including Wnt/β-catenin, Hedgehog, TGF-β (Bisson and Prowse, 2009; Chang et al., 2011; Chen et al., 2015). Targeting these pathways to eliminate PCSCs is predicted to have high therapeutic potential in prostate cancer treatment. However, delivering drugs precisely to the vicinity of the tumor to target PCSCs is still a major challenge in clinical treatment.

Recently, developed nanotechnology opens a novel avenue for drug delivery in cancer therapy. Nanotechnology is the engineering and manufacturing of materials from 1 to 100 nanometers in size in at least one dimension. Nanotechnology has been widely used in cancer diagnosis and therapy such as molecular imaging, molecular diagnosis, and targeting therapy (Toy et al., 2014; Wicki et al., 2015). For example, nanovectors are used in the clinic to facilitate the targeted delivery of imaging contrast agents for diagnosis and anticancer drugs for treatment. Nanowires and nanocantilever arrays are used for precancerous and malignant lesion detection in biological fluids (Ferrari, 2005). Some of these nanoparticle-based strategies have already been approved for clinical use, and even more are in clinical trials or in preclinical development (Zhang L. et al., 2008; Van Audenhove and Gettemans, 2016).

Here, we summarized current advances in PCSCs with a focus on their identification, origin, and maintenance signals. Furthermore, we reviewed current advances in the application of nanotechnology toward the diagnosis and therapy of prostate cancer with a specific focus on targeting PCSCs.

## Identification of PCSCs

Bonnet and Dick (1997) reported that a small subset of leukemic cells (CD34^+^CD38^-^) were capable of initiating human acute myeloid leukemia (AML) in a xenograft mouse model, this provided the first experimental evidence for the existence of cancer stem cells. Since then, although many groups have tried to identify cancer stem cells in solid tumors, it was not achieved until 2003. Al-Hajj et al. (2003) showed that CD44^+^CD24^-/low^Lineage^-^ breast cancer cells were able to initiate tumor in immunodeficient mice, which proved the existence of cancer stem cells in solid tumors. Since then, cancer stem cell markers in different solid tumors have been identified, such as brain tumors (CD133^+^) (Singh et al., 2003), lung cancer (CD133^+^) (Eramo et al., 2008), colon cancer (CD133^+^) (O’Brien et al., 2007), pancreatic cancer (CD44^+^CD24^+^ESA^+^) (Li et al., 2007), ovarian cancer (CD44^+^CD117^+^) (Zhang S. et al., 2008), hepatic carcinoma (CD45^-^CD90^+^) (Yang et al., 2008), and melanoma (ABCB5^+^) (Schatton et al., 2008).

Prostate cancer stem cells were first identified by Collins et al. (2005). Their studies showed that CD44^+^α_2_β_1_^hi^CD133^+^ cells isolated from prostate cancer patients have a high potential for self-renewal and proliferation; these cells were also able to differentiate to heterogeneous cancer cells in *ex vivo* culture (Collins et al., 2005). Since, CSCs are conceptually considered to share similar self-renewal maintenance signals with normal stem cells, researchers intended to adapt knowledge from normal stem cell studies to explain CSC regulation mechanisms. For example, Hurt et al. (2008) found that CD44^+^CD24^-^ enriched PCSC population has high level Oct3/4 and BMI-1 expression, which are critical for embryonic and quiescent adult stem cell maintenance (Masui et al., 2007; Tian et al., 2011). These isolated PCSCs have high tumorigenic and metastatic potential in immunodeficient xenograft mouse models (Hurt et al., 2008; Salvatori et al., 2012). This evidence indicated that CSCs might hijack self-renewal maintenance signals from normal stem cells during their evolution. Besides cell surface markers, some intracellular functional proteins can also be used for CSC identification. Increased aldehyde dehydrogenase (ALDH) activity is found in prostate stem/progenitor cells (Burger et al., 2009) and multiple types of CSCs, including PCSCs (Pearce et al., 2005; Ginestier et al., 2007; Jiang et al., 2009; Li et al., 2010). Prostate cancer cells with high ALDH activity showed enhanced tumorigenic and metastatic ability (van den Hoogen et al., 2010). A study identified prostate cancer cells with ALDH^+^CD44^+^α_2_β_1_^+^ phenotype could form xenograft tumors in non-obese diabetic (NOD)/SCID mice, which have impaired T and B cell lymphocyte development (SCID mutation) and deficient natural killer (NK) cell function (NOD background) (Qin et al., 2012). In addition, drug resistant genes such as ATP-binding cassette (ABC) transporter ABCG2 was used to further purify PCSCs from CD133^+^CD44^+^CD24^-^ population. These purified PCSCs have increased clone and sphere formation ability (Hirschmann-Jax et al., 2004; Pfeiffer et al., 2011; Castellon et al., 2012). Overall, these studies suggest that both self-renewal and drug resistance characteristics should be considered for CSC identification.

## The Origin of PCSCs

The origin of CSCs is still controversial. There is experimental evidence to show they could originate from normal stem cells because CSCs share similar cell surface markers with normal stem cells. For example, the first CSC study showed that CD34^+^CD38^-^ CSCs in AML share the same surface marker with hematopoietic stem cells (HSCs) (Issaad et al., 1993; Petzer et al., 1996; Bonnet and Dick, 1997). In prostate, epithelial stem cells located in the basal layer of prostate gland have cell surface markers such as CD44, α_2_β_1_, and CD133 (Collins et al., 2001; Richardson et al., 2004; Garraway et al., 2010). Interestingly, the CD44^+^α_2_β_1_^hi^CD133^+^ prostate cancer cells have been shown to be PCSCs (Collins et al., 2005). It seems that during carcinogenesis, normal prostate stem cells gain mutations in oncogenes and tumor suppressor genes that drive them to become PCSCs.

Prostate cancer stem cells can also be derived from reprograming of differentiated cells via epithelial-mesenchymal transition (EMT), in which epithelial cells lose their polarity and cell–cell adhesion and gain migratory and invasive properties of mesenchymal cells (Kong et al., 2010; Talati et al., 2015; Lee et al., 2016). Kong et al. (2010) reported that overexpression of platelet-derived growth factor D (PDGFD) resulted in the loss of epithelial markers and increasing mesenchymal markers in prostate cancer cells. These EMT transformed prostate cancer cells have enhanced clone and sphere (prostasphere)-forming ability *in vitro* and tumorigenicity in mice. They also have increased stem-cell genes such as Sox2, Nanog, Oct4, Lin28B, and Notch1 (Kong et al., 2010). Suppressing DNA methyltransferase 1 (DNMT1) by 5-azacitidine (5-Aza) in prostate cancer cells can also induce EMT and stimulate transition of PCSCs. 5-Aza treated prostate cancer cells showed enhanced CD133^+^CD44^+^ phenotype and prostasphere formation ability, and elevated expression of stem cell-related transcription factors KLF4 and Sox2 (Lee et al., 2016). Activation of Jak2-Stat5a/b signaling promotes metastasis by inducing EMT and stem cell properties in prostate cancer cells, as shown by sphere formation and expression of CSC markers BMI-1, CD44, and Sox2 (Talati et al., 2015). Recently, there is an emerging concept that EMT represents a spectrum of differentiation status ranging from fully epithelial to fully mesenchymal status (Nieto et al., 2016). It is interesting to investigate the specific EMT status that may be associated with stem cell properties. PDGFD and 5-Aza both can induce stemness of prostate cancer cells and expression of mesenchymal markers but no expression of E-cadherin (Kong et al., 2010; Lee et al., 2016); prolactin can induce the stem-like features and an intermediate EMT phenotype, with low levels of E-cadherin and concomitant mesenchymal features (Talati et al., 2015). Therefore, it seems that in prostate cancer, different degrees of EMT can be associated with stem cell properties. Overall, the EMT transformed PCSCs might have more metastatic potential compared to normal stem cell derived PCSCs. More experimental evidence is needed to fully understand the origin of PCSCs.

Prostate cancer cells can also dedifferentiate to PCSCs in bone marrow. Nearly 80–90% of patients with prostate cancer have bone metastasis (Petrylak et al., 2004; Tannock et al., 2004). Although the mechanism of the tendency to metastasize to bone is not clear, experimental evidences suggest that bone marrow may provide a microenvironment to support PCSCs, as it does for HSCs (Lymperi et al., 2010; Zhao and Li, 2015). Interestingly, disseminated tumor cells (DTCs) from prostate cancer, particularly PCSCs, can compete with HSCs to occupy bone marrow osteoblastic niche for their maintenance (Shiozawa et al., 2011). Shiozawa et al. (2011) performed a assay to recover human DTCs grown in SCID mice from bone marrow. Using this approach, they found that after intracardiac injections of non-CSC prostate cancer cells (CD133^-^CD44^-^), the CSC population (CD133^+^CD44^+^) was observed and accounted for approximately 35% of the total prostate cancer cells isolated from mouse marrow. This suggests that the enrichment of CSCs is due to the conversion of non-CSCs into CSCs. Further mechanistic analysis showed this conversion may be regulated by osteoblastic niche-derived GAS6 through the Mer/mTOR signaling (Shiozawa et al., 2016). Overall, these studies suggest PCSCs can arise from normal stem cells or from differentiated cells depending on the context.

## Self-Renewal Signaling Pathways in PCSCs

Wnt signaling is critical for embryonic stem cell transition from the pluripotent state and adult stem cell self-renewal maintenance. This raises the possibility that tightly regulated self-renewal capability in normal stem cells mediated by Wnt signal, could be hijacked by CSCs for malignant progress (Holland et al., 2013). Aberrant Wnt signaling has been reported in various tumors, including prostate cancer (Voeller et al., 1998; Chesire et al., 2000; de la Taille et al., 2003; Takebe et al., 2011). Prostate cancer patients have about 5% β-catenin activation mutation rate and this rate increases to 25–38% in metastatic and androgen-independent prostate cancer patients (Chesire and Isaacs, 2002; de la Taille et al., 2003). Two studies showed that the high incidence of β-catenin activation can induce formation of PCSCs. First, Wnt3a treatment in prostate cancer cells activated Wnt signaling and expanded PCSC numbers and increased their sphere forming ability *in vitro* (Bisson and Prowse, 2009). Second, activation of the Wnt pathway by AR79, a glycogen synthase kinase 3 (GSK-3) inhibitor, can increase the proportion of ALDH^+^CD133^+^ stem-like prostate cancer cells (Jiang et al., 2013). However, certain GSK-3 inhibitors might have varying non-specific effects, which lead to inconsistent results (Kroon et al., 2014). Therefore, targeting Wnt signaling is critical for PCSC treatment. Saikosaponin-d (SSd), a triterpenoid saponin derived from bupleurum, blocks Wnt/β-catenin signaling pathway by decreasing GSK-3β phosphorylation. SSd suppressed prostate cancer cell growth and inhibited their migration and invasion abilities. This was also accompanied by a reversal of the EMT process and inhibition of CSC phenotypes (measured by its ability to reduce tumor sphere formation and CD44 expression) (Zhong et al., 2016). In prostate cancer, PTEN is frequently mutated, which leads to activation of PI3K/Akt pathway that promotes PCSC maintenance and self-renewal (Li et al., 1997; Dubrovska et al., 2009). PI3K/Akt pathway can directly phosphorylate β-catenin at serine 552 to induce its nuclear localization, which leads to activation of Wnt signaling (Fang et al., 2007; He et al., 2007). Akt can also activate Wnt signaling through phosphorylation and inactivation of GSK-3β (Sharma et al., 2002). However, this mechanism is not universally supported. In a traumatic brain injury rat model, the peak time points of Akt and GSK-3β phosphorylation are not synchronous, suggesting GSK-3β may not be phosphorylated by Akt pathway (Zhao et al., 2012). Moreover, simultaneous activation of Wnt/β-catenin and PI3K/Akt signaling is required to drive self-renewal and expansion of HSCs (Perry et al., 2011). These findings indicate that Wnt/β-catenin and PI3K/Akt signaling can cooperatively promote CSC self-renewal. Thus, how to target these two pathways simultaneously may be critical to eliminate PCSCs.

Androgen signaling controls the growth of prostate gland and AR plays important roles throughout the various stages of prostate cancer (Augello et al., 2014). Interestingly, the expression and function of AR in PCSCs are still debatable. In many reported PCSC populations, AR expression is often low or undetectable. For example, the CD44^+^α_2_β_1_^+^CD133^+^ cells purified from human prostate tumor samples (Collins et al., 2005), the CD44^+^ cells in several prostate cancer xenografts (Patrawala et al., 2006), and the BCRP^+^ putative PCSCs (Huss et al., 2005) are all AR^-^. However, some studies show conflicting data. It was reported that the CD133^+^ cancer-initiating population and CD44^+^CD24^-^ putative PCSCs in prostate cancer cell lines are AR^+^ (Sharifi et al., 2008; Vander Griend et al., 2008). Deng and Tang provide a hypothesis that PCSCs in primary and untreated tumors and models are mainly AR^-^, whereas PCSCs in castration resistant tumors could be either AR^+^ or AR^-/lo^ (Deng and Tang, 2015). Interestingly, androgen signaling can interact with Wnt signaling and PI3K/Akt signaling at multiple levels (Terry et al., 2006; Lee et al., 2015). β-catenin can directly bind to ligand-engaged AR protein to promote its transcription activity. This binding can also facilitate the translocation of β-catenin into the nucleus (Truica et al., 2000; Mulholland et al., 2002; Yang et al., 2002). GSK-3β phosphorylates AR, thereby inhibits AR-driven transcription, which can be abrogated by the GSK-3 inhibitor LiCl (Salas et al., 2004). Human AR gene promoter contains LEF-1/TCF binding elements and activation of Wnt signaling upregulates AR transcription. In contrast, Wnt activation suppresses AR protein level by increasing phosphorylation of Akt and its downstream target MDM2, which promotes degradation of AR protein (Yang et al., 2006). Moreover, AR inhibition can activate Akt signaling by reducing levels of AKT phosphatase PHLPP in prostate PTEN-deficient murine prostate cancer model and in human prostate cancer xenografts (Carver et al., 2011). Overall, these findings indicate that androgen signaling has complex crosstalk with Wnt/β-catenin and PI3K/Akt signaling, and may enable prostate cancer cell stemness through Wnt/β-catenin and PI3K/Akt signaling.

The importance of Wnt/β-catenin signaling in tumors has spurred the development of inhibitors for cancer therapy. Cell-line studies have suggested some Wnt inhibitors exert inhibitory effects on prostate cancer cell proliferation and several Wnt inhibitors have been proven to be effective at inhibiting PCSCs. PKF118-310 suppresses prostate cancer cell growth by inhibiting β-catenin and TCF complex mediated transcription activation (Lepourcelet et al., 2004; Lu et al., 2009). 3289–8625 suppresses prostate cancer cell proliferation and reduces β-catenin level by inhibiting DVL-1 which links frizzled receptors and downstream signals (Grandy et al., 2009). Pyrvinium inhibits AR dependent gene expression and prostate cancer cell growth, which may result from its inhibitory effect on Wnt signaling through potentiating casein kinase 1α (CK1α) kinase activity (Jones et al., 2009; Thorne et al., 2010). Additionally, a study showed niclosamide, a drug used for the treatment of tapeworm, suppresses prostate cancer cell growth by inducing degradation of the Wnt receptor LRP6 (Lu et al., 2011). Importantly, DKK1 and sFRP2, two inhibitors that block Wnt signaling by binding to Wnt receptor LRP5/6 (DKK1) or Wnt proteins (sFRP2) (Kawano and Kypta, 2003), significantly inhibit the self-renewal capacity of PCSCs as evidenced by their ability to decrease prostasphere size and formation (Bisson and Prowse, 2009).

Several other signaling pathways are also implicated in PCSC regulation. Sanchez et al. (2004) found that sonic hedgehog (SHH) pathway components, such as GLI1, PTCH1, and SHH are upregulated in human prostate cancer tissues compared with normal prostatic epithelia. SHH signaling can be activated by androgen deprivation (Chen et al., 2009). Activation of SHH signaling supports androgen independent cell growth in a low androgen environment and enhances therapy resistance by increasing the level of ABC transporter (Chen et al., 2010; Statkiewicz et al., 2014). Blocking SHH pathway with an anti-SHH antibody or cyclopamine, a SMOH inhibitor, suppressed prostate cell proliferation (Chen et al., 2002; Sanchez et al., 2004). Overexpression of hedgehog leads to the formation of PCSCs with increased metastasizing potential (Chang et al., 2011). Darinaparsin, an organic arsenical compound with potent antineoplastic ability (Mann et al., 2009), and Genistein, an isoflavone with inhibitory effect on tyrosine kinases and topoisomerase-II (Salti et al., 2000; Qin et al., 2015), can both inhibit stemness of PCSCs and reduce tumor formation in xenograft models through targeting SHH signaling pathway (Zhang et al., 2012; Bansal et al., 2015).

Prostate carcinoma have high levels of TGF-β and TGF-β receptor expression (Cardillo et al., 2000). During prostate cancer progression, TGF-β plays an inconsistent role. During tumor initiation, TGF-β suppresses tumor growth by inducing apoptosis (Diener et al., 2010), while during tumor progression TGF-β induces EMT for invasion and metastasis (Moustakas and Heldin, 2016). This phenomenon is known as the TGF-β paradox (Tian and Schiemann, 2009). Activation of TGF-β signal expanded the CD44^+^CD24^-^ population in prostate cancer cells through downregulating poly r(C) binding protein (PCBP)-1 (Chen et al., 2015), which suggested that TGF-β might regulate PCSC maintenance.

Non-coding RNAs are also involved in regulation of PCSCs stemness. Long non-coding RNA (lncRNA) H19 is highly expressed in PCSCs and knockdown of H19 decreases the colony-forming efficiency and reduces the expression of stem-cell genes (Oct4, Sox2, and Notch1). On the other hand, overexpression of H19 favors stemness of PCSCs (Bauderlique-Le Roy et al., 2015). lncRNA Hotair works synchronously with PRC2 to transcriptionally downregulate AR, leading to the increase of the CD133^+^ stem cell population (Li et al., 2015). In addition, microRNAs (miRNAs) are shown to regulate PCSCs through several stemness-related pathways such as Wnt, Akt, and TGF-β pathway. Increasing β-catenin expression through decreasing miRNA-320 in prostate cancer cells significantly increased their tumor spheres formation and clonogenic capacity, along with an increase in chemotherapy resistance *in vitro* and tumor growth in prostate cancer xenografts (Hsieh et al., 2013). Reduced miR-708 expression enhances PCSC stemness by upregulating AKT2, while re-expressing miR-708 suppresses the clonogenicity *in vitro* and leads to tumor regression in prostate cancer xenografts (Saini et al., 2012). MiR-128 overexpression in prostate cancer cells inhibits clonogenic and sphere-forming activities by decreasing stem cell regulatory factors BMI-1, Nanog, and TGFβR1 (Jin et al., 2014).

Conceivably, these self-renewal signaling pathways could serve as PCSC therapeutic targets in the future. However, most of the inhibitors against self-renewal pathways have clinical side effects and toxicities, which limit their clinical use. Since somatic stem cell homeostatic and regenerative processes after injury also rely on the self-renewal pathways for tissue regeneration and stem cells maintenance, inhibitors targeting these pathways may cause systemic toxicities (Pattabiraman and Weinberg, 2014). For example, it is well-known that Wnt signaling is essential for the regulation and homeostasis of intestinal stem cells (Pinto et al., 2003). Wnt inhibitors may lead to a depletion of normal intestinal stem cells (Kahn, 2014). Nanotechnology-based drug delivery systems can greatly improve this situation by increasing targeting specificity and reducing toxicities through restriction of drugs to the immediate vicinity of the tumor.

## Application of Nanotechnology in Prostate Cancer

Recently, nanotechnology has been extensively explored in biomedical field to facilitate diagnosis and drug delivery for cancer treatment (Wu et al., 2010; Liao et al., 2011). Nanoparticles are small in size but with large surface-to-volume ratios allowing attachment of various molecules such as drugs and antibodies, which makes them suitable for medical use (Whitesides et al., 1991). Currently, prostate cancer diagnosis methods in the clinic include biochemical assays, digital rectal examination, transrectal ultrasonography, and biopsy. Biochemical assays are usually the first step for prostate cancer screening that examines the serum level of prostate specific antigen (PSA) (Catalona et al., 1991). PSA is a serine protease secreted by normal and malignant prostatic epithelium into seminal fluid, with minor amounts leaking into circulation in normal state, but increased amounts are observed in prostatic cancer (Stenman et al., 1999). According to the guidelines approved by the US Food and Drug Administration (FDA), a concentration of PSA > 4 ng/mL is considered as the gold standard of prostate cancer in initial screening. However, currently used enzyme-linked immunosorbent assay (ELISA) detection method for PSA shows poor sensitivity and specificity, with approximately 70% false-positive rate (Catalona et al., 1991; Bretton, 1994; Kang B.J. et al., 2015). Various nanomaterials with unique properties such as strong electronic, optic, and magnetic properties have been developed for PSA detection with better sensitivity. Among these nanotechnology-based bioassays, the most popular method is the electrochemical assay. In this assay, PSA captured by specific antibody alters the current that runs through carbon nanotubes, which gives this assay a higher sensitivity and a quicker speed than the standard ELISA method (Panini et al., 2008; Kim et al., 2009; Pandey et al., 2012; Huang et al., 2013; Salimi et al., 2013; Wang et al., 2013). Gold nanoparticles, with high surface area to volume ratio allowing more antibodies loading, can significantly improved PSA detection sensitivity in both serum (Thaxton et al., 2009) and urine samples (Yuhi et al., 2006). Besides PSA, other biomarkers such as prostate specific membrane antigen (PSMA), PF-4, IL-6, and ANXA3 can also be used for prostate cancer diagnosis, which have been tested using nanomaterials (Chikkaveeraiah et al., 2009; Kim et al., 2013). miRNAs are expressed in a tissue- and function-specific manner and are protected from nuclease degradation in the bloodstream. This makes them new candidate biomarkers for detecting cancers (Lu et al., 2005; Mitchell et al., 2008). MiR-141, with an elevated level in the blood of patients having metastatic prostate cancer (Mitchell et al., 2008), can be detected by a polymer-based nanomaterial (Tran et al., 2013). Another interesting study used spherical gold nanoparticle-nucleic acid conjugates to develop a microRNA array system for detection of microRNA profiles in prostate cancer samples. Through this system, they found several differentially expressed microRNAs (miR-200c, -21, -210, -205, -20a, -143^∗^, -143, and -16) that can be used as biomarkers (Alhasan et al., 2012).

Despite many chemotherapeutic agents show promising results in preclinical settings, their application in clinic often meets limitations largely due to inefficient bioavailability. Nanotechnology can improve drug bioavailability by developing a variety of nanoparticles that encapsulate anti-tumor drugs and release drugs in a controlled and time-dependent manner. Green tea polyphenol epigallocatechin-3-gallate (EGCG) can induce apoptosis of prostate cancer cells (Stuart et al., 2006). A polylactic acid-polyethylene glycol nanoparticle that encapsulated EGCG showed better pro-apoptotic and angiogenesis-inhibitory effects *in vitro* and larger inhibitory effect on prostate tumor growth in xenograft mice model than the non-encapsulated EGCG (Siddiqui et al., 2009). Camptothecin (CPT) is a pentacyclic alkaloid with a wide spectrum of anti-cancer activities, but is poorly soluble and has a fast degradation rate. CPT encapsulated β-cyclodextrin-nanosponges has been reported to improve the inhibitory effect on prostate cancer cell growth (Gigliotti et al., 2016). Besides improving bioavailability, nanotechnology can also specifically deliver chemotherapeutic agents to cancer cells without damaging the healthy cells. This targeted delivery is achieved by conjugating antibodies against tumor antigens to nanoparticles. In prostate cancer, PSMA and prostate stem cell antigen (PSCA) are the mostly used conjugated antibodies, both of which are highly expressed in prostate cancer cells (Reiter et al., 1998; Ghosh and Heston, 2004). The unique magnetic properties of some nanomaterials can be utilized in real-time monitoring of drug distribution. Nanoparticles that contain anti-tumor drugs and targeting ligands/antibodies can be coupled with the real-time imaging for the quantification of targeting efficiency. These reagents are defined as theranostic nanomedicine (Cherian et al., 2014). For example, PSMA targeted and PSCA targeted docetaxel-loaded superparamagnetic iron oxide (SPIO) nanoparticles can be efficiently internalized in prostate cancer cells and exhibit a higher inhibitory effect on cell survival compared with free docetaxel in prostate cancer cells. The distribution of these nanoparticles in cells can be visualized because SPIO is a kind of magnetic resonance imaging (MRI) contrast agent (Ling et al., 2011; Nagesh et al., 2016). Abdalla et al. (2011) engineered an iron oxide nanoparticle that targeted the drug noscapine (Nos) to tumors using urokinase plasminogen activator (uPA), a natural ligand for uPA receptor (uPAR) that is highly expressed by prostate cancer cells. The uPAR-targeted Nos-loaded iron oxide nanoparticles enhance the inhibitory effect of noscapine on prostate cancer cell growth and maintain their T2 MRI contrast effect upon internalization into tumor cells (Abdalla et al., 2011).

To date, nanotechnology has been applied to destroy PCSCs (**Table 1**). Nanoparticles loaded with self-renewal pathway inhibitors are designed in order to inhibit stemness of PCSCs. The clinical use of cyclopamine, a hedgehog inhibitor, is limited by its high hydrophobicity, systemic toxicity and poor pharmacokinetics (Lipinski et al., 2008). *N*-(2-hydroxypropyl) methacrylamide (HPMA) copolymers are great drug carriers with the advantage of increased solubility, prolonging circulation time and improved pharmacokinetic profiles of small molecule drugs (Kopecek and Kopeckova, 2010; Zhou and Kopecek, 2013). HPMA copolymer-cyclopamine conjugate treatment significantly decreased prostasphere forming capacity and percentage of CD133^+^ PCSC enriched population in PC3 and RC-92a/hTERT prostate cancer cells. RC-92a/hTERT cells are human prostate cancer epithelial cells transduced to express human telomerase reverse transcriptase, and exhibit high levels of CD133 (Miki et al., 2007). *In vivo* experiment showed that HPMA copolymer-cyclopamine conjugate administration reduced tumor volume in PC3 tumor xenograft nude mice. Moreover, combination of HPMA copolymer-cyclopamine conjugate and HPMA copolymer-docetaxel conjugate led to significantly reduced tumor volume over single drug administration (Zhou et al., 2012, 2013). HPMA copolymer-GDC-0980 (PI3K/mTOR inhibitor) conjugate treatment can also decrease the percentage of CD133^+^ cells and the number of prostaspheres in PC3 cells. In PC3 tumor xenograft model, administration of HPMA copolymer-GDC-0980 conjugate could prolong survival slightly, and combination use of HPMA copolymer-GDC-0980 conjugate and HPMA copolymer-docetaxel conjugate led to significantly prolonged survival compared with either of the single treatments (Zhou et al., 2015). Yang et al. (2016) synthesized poly(ethylene glycol)-block-poly(2-methyl-2-carboxyl-propylene carbonate) (mPEG-b-PCC) for loading cyclopamine and paclitaxel, respectively. Both the cyclopamine and paclitaxel loaded nanoparticles can release drugs slowly and inhibit colony-forming ability of paclitaxel resistant PC3 cells. Administration of either of the two nanoparticles to PC3 tumor xenograft nude mice can lower tumor growth. Significant tumor inhibition was observed in mice treated with the combination of cyclopamine and paclitaxel loaded nanoparticles (Yang et al., 2016). These studies suggest combination therapy targeting both CSCs and bulk tumor cells is a promising approach to improve the therapeutic benefit against prostate cancer. *Cis-*dichlorodiamminoplatinum (II) (CDDP) is a highly effective anti-tumor agent toward a variety of tumor types. Jafari Malek et al. (2014) generated CDDP loaded glyconanoparticles using hyaluronic acid (HA), the endogenous substrate for CD44. These CDDP loaded glyconanoparticles led to a significant reduction of clonogenicity and sphere formation capacity of prostate cancer DU145 and PC3 cells (Jafari Malek et al., 2014). Alongside the drugs conjugated to nanoparticles, some materials themselves can exert anti-tumor functions. An interesting study found graphene oxide effectively inhibited sphere formation not only in PC3 prostate cancer cells, but also in SKOV3 ovarian cancer cells, U87 glioblastoma cells, A549 lung cancer cells, and MIA-PaCa-2 pancreatic cancer cells, highlighting its efficacy against CSCs across different cancer types. Graphene oxide exerts this effect by inducing CSC differentiation through blocking several key signaling pathways including Wnt, Notch, and STAT (Fiorillo et al., 2015).

**Table 1:** Summary of nanosystems used in PCSC research.

Nanomaterial	Drug	Experimental subject	Effect	Reference
HPMA	Cyclopamine	RC-92a/hTERT and PC3 cell lines	*In vitro:* sphere-forming capacity↓ percentage of CD133^+^ population↓	Zhou et al., 2012, 2013
		PC3 tumor xenograft nude mice	*In vivo*: tumor growth↓	
HPMA	GDC-0980	PC3 cell line	*In vitro:* sphere-forming capacity↓ percentage of CD133^+^ population↓	Zhou et al., 2015
		PC3 tumor xenograft nude mice	*In vivo*: mouse survival↑	
mPEG-b-PCC	Cyclopamine	Paclitaxel resistant PC3 cell line	*In vitro:* colony-forming capacity↓	Yang et al., 2016
	Paclitaxel	PC3 tumor xenograft nude mice	*In vivo:* tumor growth↓	
HA	CDDP	DU145 and PC3 cells lines	*In vitro:* sphere-forming capacity↓ colony-forming capacity↓	Jafari Malek et al., 2014
GO		PC3 cell line	*In vitro*: sphere-forming capacity↓	Fiorillo et al., 2015


*CDDP, *cis*-dichlorodiamminoplatinum (II); GO, graphene oxide; HA, hyaluronic acid; HPMA, *N*-(2-hydroxypropyl) methacrylamide; mPEG-b-PCC, poly(ethylene glycol)-block-poly(2-methyl-2-carboxyl-propylene carbonate).*

## Challenges and Perspectives

There is a significant and rapid advancement in our knowledge of PCSCs and their role in prostate cancer initiation and progression. We can target PCSCs through their self-renewal pathways, such as Wnt signaling. Several inhibitors such as DKK1 and sFRP2 for Wnt signaling are effective at inhibiting PCSC self-renewal (Bisson and Prowse, 2009). However, their potential adverse effects on normal stem cell self-renewal and tissue homeostasis are a serious concern. More effective drug delivery system is urgently needed. The application of nanotechnology-based drug delivery such as nanoparticle capsules can improve PCSC targeting specificity and reduce side effects by restricting drugs to tumors and their surrounding areas.

Targeted delivery of drugs to CSCs without damaging normal stem cells is challenging because of shared cell surface markers. A variety of drug-loaded nanoparticles conjugated with antibodies to these markers (CD44, CD133, and ABCG2) have been developed that improve the drug delivery efficiency to CSCs in colon cancer (Bourseau-Guilmain et al., 2012), breast cancer (Swaminathan et al., 2013), and multiple myeloma (Yang et al., 2014). Some nanomaterials with photo-thermal properties, such as single-walled carbon nanotubes (SWNTs), can be used for thermal destruction of glioblastoma stem-like cells when conjugated with CD133 antibody (Wang et al., 2011). Nanomaterials like HA, which has high CD44-binding efficacy, can deliver drugs to CD44-expressing CSCs (Wei et al., 2013). However, it is unknown whether these CSC-targeted nanoparticles will have toxic effects on normal stem cells. One possible solution is to identify markers with specific expression on CSCs but not on normal stem cells.

Since, CSCs and normal stem cells often share the same self-renewal pathways, identifying and targeting the key signaling involved in CSCs but not in normal stem cells is a promising strategy. Kang X. et al. (2015) found that Leukocyte-associated immunoglobulin-like receptor 1 (LAIR1) deficiency exhausts mouse AML stem cells, but does not affect normal hematopoiesis. This discovery provides hope that there may likewise be similar pathways in PCSCs. The hypoxia-inducible factor (HIF) pathway may be one of such pathways in prostate cancer. HIF signaling is elevated in PCSC population and promotes stemness and self-renewal of PCSCs (Ma et al., 2011; Marhold et al., 2015). Considering hypoxia often presents in the tumor microenvironment instead of the normal state, targeting HIF signaling may inhibit PCSCs without damaging normal stem cells.

**FIGURE 1:**
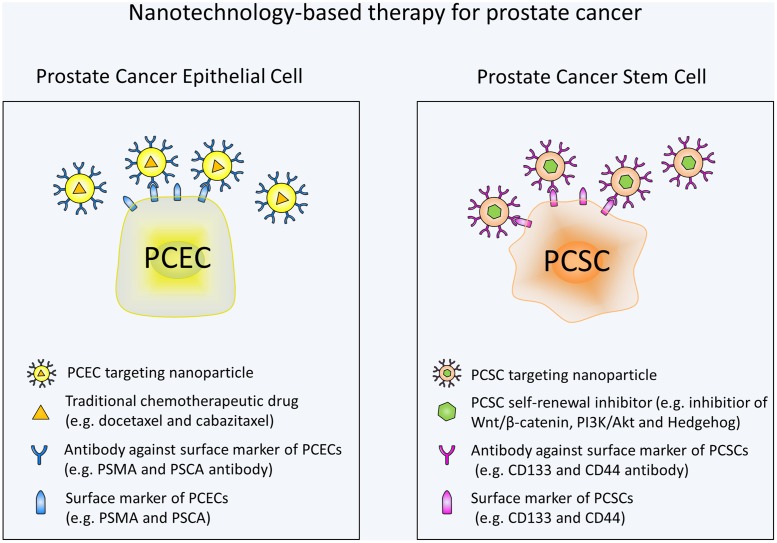
**Schematic illustration of nanotechnology-based therapy for prostate cancer.** Prostate cancer epithelial cell (PCEC) targeting nanoparticles are conjugated with antibodies against PCEC surface markers and contain traditional chemotherapeutic drugs. PCSC targeting nanoparticles are conjugated with antibodies against PCSC surface markers and contain self-renewal signaling pathway inhibitors. Combination use of the nanotechnology-based standard chemotherapy and PCSC specific chemotherapy can eradicate the bulk of the tumor cells and PCSCs at the same time, thus may be the most efficacious treatment for prostate cancer.

Overall, there has been much advancement in the field of nanotechnology for prostate cancer treatment. Various approaches have been developed to specifically target PCSCs. In the future, more nanotechnology-based therapeutic strategies are urgently needed to target self-renewal pathways of PCSCs. Wnt signaling with its critical role in PCSCs and existence of a variety of small-molecule inhibitors is an attractive target. Moreover, additional studies are still needed to investigate the specific markers and pathways involved in PCSCs. By targeting these markers and pathways, nanoparticles may avoid the toxic effects on normal stem cells. It should also be noted that targeting the PCSC alone may not be enough to eliminate tumor and combination of a standard chemotherapy and a PCSC specific chemotherapy may be the most efficacious treatment for prostate cancer (**Figure 1**). Based on the studies mentioned in our review, it is apparent that nanotechnology-based methods holds great potential for the targeted destruction of PCSCs and may lead to significant patient benefit.

## Author Contributions

WQ and YZ wrote the manuscript. B-ZQ and MZ provided critical comments and revised the manuscript.

## Conflict of Interest Statement

The authors declare that the research was conducted in the absence of any commercial or financial relationships that could be construed as a potential conflict of interest.
